# Copper Sulfide Nanorod-Embedded Urinary Catheter with Hydrophobicity and Photothermal Sterilization

**DOI:** 10.3390/ijms252111440

**Published:** 2024-10-24

**Authors:** Muhammad Saukani, Chien-Hung Lai, Chinmaya Mutalik, Dyah Ika Krisnawati, Hsiu-Yi Chu, Tsung-Rong Kuo

**Affiliations:** 1 International Ph.D. Program in Biomedical Engineering, College of Biomedical Engineering, Taipei Medical University, Taipei 11031, Taiwan; 2 Department of Mechanical Engineering, Faculty of Engineering, Universitas Islam Kalimantan MAB, Banjarmasin 70124, Kalimantan Selatan, Indonesia; 3 Department of Physical Medicine and Rehabilitation, School of Medicine, College of Medicine, Taipei Medical University, Taipei 11031, Taiwan; chlai@tmu.edu.tw; 4 Department of Physical Medicine and Rehabilitation, Taipei Medical University Hospital, Taipei 11031, Taiwan; 5 Taipei Neuroscience Institute, Taipei Medical University, Taipei 11031, Taiwan; 6 Graduate Institute of Nanomedicine and Medical Engineering, College of Biomedical Engineering, Taipei Medical University, Taipei 11031, Taiwan; cm121193@tmu.edu.tw; 7 Department of Nursing, Faculty of Nursing and Midwifery, Universitas Nahdlatul Ulama Surabaya, Surabaya 60237, East Java, Indonesia; dyahkrisna77@gmail.com; 8 Graduate Institute of Biomedical Materials and Tissue Engineering, College of Biomedical Engineering, Taipei Medical University, Taipei 11031, Taiwan; d825111001@tmu.edu.tw

**Keywords:** copper sulfide, urinary catheter, photothermal therapy, hydrophobicity, biocompatibility, anti-cell adhesion

## Abstract

The high prevalence of catheter-associated urinary tract infections (CAUTIs) is causing significant concern in healthcare systems. Antibacterial urinary catheters have been developed to prevent CAUTIs in clinical application. In this work, a copper sulfide nanorod (CuS NR)-embedded urinary catheter (CuS/UC) was designed as an antibacterial urinary catheter with photothermal sterilization. The CuS NRs with low cytotoxicity were synthesized via the hydrothermal method. The CuS NRs were embedded into urinary catheters at different weight percentages. The CuS/UC exhibited homogenous surface roughness, low wettability, hydrophobicity, and low adhesiveness, promoting minimal interaction with bacteria and healthy cells. Under near-infrared (NIR) laser irradiation, the 0.8 weight percentage of CuS NRs in the urinary catheter (0.8CuS/UC) reached a temperature of 67.4 °C, demonstrating its photothermal antibacterial activity and suitability for catheter sterilization. Agar plate test verified that CuS/UCs exhibited a superior photothermal antibacterial activity against both Gram-negative *Escherichia coli* (*E. coli*) and Gram-positive *Streptococcus aureus* (*S. aureus*). Moreover, the 0.8CuS/UC exhibited excellent biocompatibility and anti-cell adhesion properties. The 0.8CuS/UC with photothermal performance, excellent biocompatibility, and anti-cell adhesion properties demonstrated its potential as a photothermal antibacterial catheter for clinical applications.

## 1. Introduction

Over 1 million cases of catheter infections in the United States and nearly 80% of infectious diseases globally are caused by antimicrobial resistance and catheter-associated infections [1]. Liquids and injections that are vitally required during medical therapy are frequently administered via biomedical catheters, such as intravascular and urine catheters. One of the most frequent sources of infection is catheter-associated urinary tract infections (CAUTIs) due to bacterial adhesion. An indwelling urinary catheter will be present in about 12–16% of adults with chronic conditions at some point during their hospital stay, and for every day a catheter is in place, the chance of contracting a bacterial infection and developing antibiotic resistance increases by 3–7% [2,3,4,5,6]. Bacterial biofilms and bacterial resistance are major problems in the development of urinary catheter infections. Based on the mechanisms of biofilm formation, there are five strategies to overcome these problems, including biocide-releasing urinary catheters (BRUCs), contact killing, modifications of the catheter surface to avoid early adherence, biofilm disruption, and the use of a benign bacterial biofilm to suppress pathogen colonization [7,8,9,10,11]. Among these strategies, BRUCs and surface modifications of urinary catheters are popular strategies chosen to prevent bacterial colonization using antibiotics, nanoparticles, and metal ions [12,13,14,15]. The commonly used biocide-releasing agents are silver-based materials because of their antimicrobial ability and United States Food and Drug Administration approval as catheter coatings [16,17]. However, due to a lack of control, silver ion leakage and toxicity can endanger the sensitive urethral mucosa. For both effective prevention and sterilization, an alternative approach for the development of antibacterial catheters is an urgent requirement to fight CAUTIs.

Nanomaterials with superior characteristics of structural complexities and optical features have offered an opportunity to explore further and enhance their antibacterial activities, potentially paving the way for clinical applications [18]. Great achievements of the nanomaterials have been demonstrated for antibacterial applications based on mechanisms such as metal ion release, photodynamic therapy (PDT), and photothermal therapy (PTT) [19,20,21,22]. For instance, urinary catheter combined with silver nanoparticle-decorated amphiphilic carbonaceous particles exhibited a continuous release of silver ions over a period of 30 days, resulting in a potent biocidal activity and effective prevention of biofilm formation both in vitro and in an in vivo rabbit model [17]. Metallic and semiconducting molybdenum disulfide (MoS_2_) nanosheets have revealed photodynamic antibacterial activity for the generation of reactive oxygen species against *E. coli* [23]. Gold nanorods and gold nanobipyramids have been employed as photothermal agents for the noninvasive therapy of *E. coli*, exhibiting photothermal capability and reversible laser response under near-infrared (NIR) laser irradiation [24]. A hybrid nanocomposite of polyurethane, polyethylene glycol, and gold nanorods has effectively combated multidrug-resistant bacteria under NIR light irradiation [25]. Nanoantibiotics based on polypyrrole, exhibiting superior photothermal effects in the NIR-II region (around 1064 nm), have been engineered to effectively treat multidrug-resistant bacterial infections [26]. Photothermal therapy, recognized for its potential in antibacterial application, has been extensively demonstrated as an effective antibacterial approach. With the uses of light-absorbing nanomaterials to generate heat, photothermal therapy can selectively target and kill bacteria [27,28,29]. Photothermal therapy has shown promise for combating bacterial infections while minimizing the risk of resistance development.

Transition metals have diverse chemical properties, allowing for the design of nanomaterials with tailored characteristics suited for specific biomedical applications [30]. Transition metal-based nanomaterials (TMNs) with unique properties, such as size, shape, and surface chemistry, can be precisely controlled, enabling customization for various biomedical functions like drug delivery, imaging, and therapy [31,32,33,34]. Moreover, many of TMNs have exhibited excellent biocompatibility, reducing the risk of toxicity and adverse reactions in biological systems [35,36,37]. TMNs have also revealed high stability under physiological conditions, ensuring their functionality and integrity during biomedical applications [38,39]. Recent demonstrations have revealed the highly effective photothermal effects of TMNs in reducing bacterial resistance and outbreaks. For example, experiments and density functional theory simulations have elucidated the NIR-based electronic structure–activity relationship of metallic and semiconducting MoS_2_ nanosheets regarding the influence of photothermal performance on bacteria eradication [40]. Dong et al. reported that the combination between an antimicrobial peptide, daptomysin (Dap), in an antimicrobial nano-system comprising Dap@Au/Ag nanorods was achieved as an innovative agent against *methicillin-resistant Staphylococcus aureus* upon exposure to NIR laser irradiation, leveraging the excellent photothermal effect of gold nanorods [41]. Different structures of copper sulfide (CuS), comprising nanosheets and nanoparticles, have been synthesized and employed for antibacterial purposes, showcasing the notable degradation of *E. coli* upon NIR laser exposure, mainly attributed to their photothermal killing effects [42]. While transition metal-based nanomaterials have shown success as photothermal antibacterial agents, their utilization in antibacterial urinary catheters with photothermal therapy remains limited, highlighting the need for further investigation and development.

In this study, TMNs of CuS nanorods (NRs) were synthesized via the hydrothermal approach. The synthesized CuS NRs were comprehensively characterized through scanning electron microscopy (SEM), transmission electron microscopy (TEM), X-ray diffraction (XRD), Raman spectroscopy, X-ray photoelectron spectroscopy (XPS), ultraviolet–visible–near infrared (UV-Vis-NIR) spectroscopy, and Fourier transform infrared (FTIR) spectroscopy. The cytotoxicity test of CuS NRs on Vero cells was assessed using the methyl thiazolyl tetrazolium (MTT) assay. The CuS NRs were embedded into urinary catheters with various weight percentages of CuS NRs, including 0.0% (UC), 0.1% (0.1CuS/UC), 0.3% (0.3CuS/UC), 0.5% (0.5CuS/UC), and 0.8% (0.8CuS/UC). The surface homogeneity of CuS/UC was studied by atomic force microscopy (AFM). The hydrophobicity of CuS/UC was examined by the contact angle. The photothermal performance of CuS/UC was investigated by irradiation with an 808 nm NIR laser at the power densities of 0.5, 1.0, and 1.5 W/cm^2^. The photothermal antibacterial activity of CuS/UC was evaluated with NIR laser irradiation based on the bacterial growth curve. Furthermore, the agar plate test was applied to confirm the photothermal antibacterial activity of CuS/UC. The biocompatibility and anti-cell adhesion of CuS/UC were investigated by in vitro Vero cells.

## 2. Results and Discussion

### 2.1. Structural and Optical Characterizations of CuS NRs

**Figure 1:**
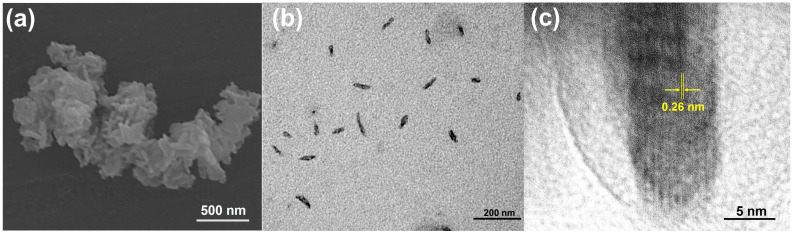
(**a**) SEM, (**b**) TEM, and (**c**) HR-TEM images of CuS NRs.

The simple hydrothermal method was followed by the ultrasonication process of a dry precipitate powder of CuS in an n-hexane solution, successfully synthesizing CuS NRs. The structural properties of CuS NRs were confirmed by SEM, TEM, and HR-TEM. As shown in the SEM image of Figure 1a, the rod structure of CuS NRs was observed. Furthermore, an energy-dispersive X-ray (EDX) analysis of CuS NRs was conducted to confirm their composition. As shown in the Supporting Information of Figure S1, the EDX spectrum indicated that the CuS NRs were composed of copper and sulfur. The elemental mapping analysis of CuS NRs also confirmed the composition of copper and sulfur. After the calculations from the EDX spectrum, CuS NRs were found to be composed of copper (Cu, 65.79 wt%) and sulfur (S, 34.21 wt%). The atomic ratio of S:Cu of CuS NRs was 1.03:1.04, in accordance with the chemical formula of CuS. Moreover, the morphology of CuS NRs was examined by TEM. As shown in the TEM image of Figure 1b, the rod structure of CuS NRs was confirmed. In Figure 1b, the length and diameter of CuS NRs were, respectively, calculated to be 55.01 ± 10.88 nm and 21.35 ± 2.51 nm using the ImageJ 1.54g software. Additionally, as shown in the HR-TEM image of Figure 1c, CuS NRs had a lattice spacing of 0.26 nm, indicating a hexagonal arrangement of the Cu and S atoms [43,44]. For CuS, the rod-like shape can be attributed to the preferred direction of crystal development and crystal nucleation. Interactions among solvent molecules and the surfaces of nanoparticles in the system significantly impact their morphology and size, making solvent polarity crucial. The kinetics of the solvents govern the pace of particle–particle interactions and aggregation during the process. Generally, in the absence of a stabilizing agent, the solvent composition predominantly determines the potency of the electrical charge of the double layer, which controls crystal growth. Here, the polarity index of the solvent is essential. As a result, the zeta potential of the particles increases, causing them to repel one another, preventing aggregation. This may explain the various shapes of CuS produced by ethanol-based synthesis. The subsequent sonication in hexane resulted in CuS NRs with a rod-like shape [45,46,47,48].

**Figure 2:**
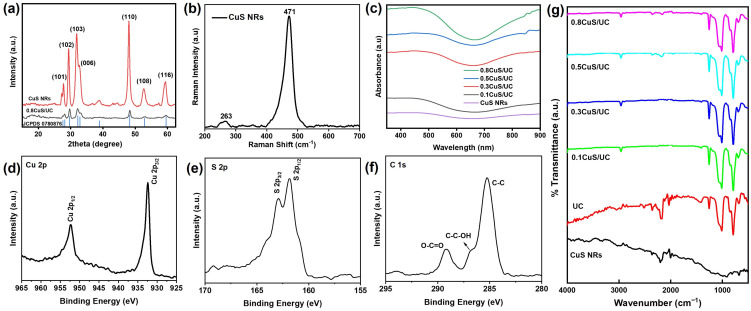
(**a**) XRD patterns of CuS NRs and 0.8CuS/UC. (**b**) Raman spectrum of CuS NRs. (**c**) UV-Vis-NIR spectra of CuS NRs, 0.1CuS/UC, 0.3CuS/UC, 0.5CuS/UC, and 0.8CuS/UC. XPS spectrum of CuS NRs of (**d**) Cu 2p, (**e**) S 2p, and (**f**) C 1s. (**g**) FTIR spectra of CuS NRs, UC, 0.1CuS/UC, 0.3CuS/UC, 0.5CuS/UC, and 0.8CuS/UC.

The structural properties of CuS NRs and 0.8CuS/UC were further studied by XRD in order to determine whether the properties of CuS NRs were altered after being introduced to prepare the CuS/UC. In Figure 2a, the diffraction peaks of CuS NRs were observed at the 2θ angles of 27.79°, 29.40°, 31.85°, 32.82°, 49.10°, 53.38°, and 59.79°, corresponding to the Miller indices (101), (102), (103), (006), (110), (108), and (116). These peaks indicate a hexagonal arrangement of Cu and S atoms in the lattice, as confirmed by the XRD analysis [49,50]. The XRD pattern of 0.8CuS/UC displayed a diffraction pattern similar to the single phase of CuS, which matched PDF number 01-078-0876. The XRD analysis results indicate that the CuS NR structure remained unaffected in the CuS/UC. Moreover, Raman spectroscopy was also used to confirm the production of CuS NRs for optical characterization. As shown in Figure 2b, one Raman peak at 263 cm^−1^ was correlated with the Cu-S stretching phonon mode of CuS NRs. The sharp Raman peak at 471 cm^−1^ was correlated with S-S stretching phonon modes. These Raman signals verified the production of CuS NRs [51,52,53]. Additionally, UV-Vis-NIR spectroscopy was used to validate the optical characteristics of CuS NRs and CuS/UC at various concentrations. As shown in Figure 2c, CuS NRs, 0.1CuS/UC, 0.3CuS/UC, 0.5CuS/UC, and 0.8CuS/UC exhibited an absorption band at 808 nm due to the d-d transition of Cu(II) ions in a tetrahedral stereochemistry, which is typical for copper ions in the CuS network [54]. Moreover, an XPS analysis was applied to investigate the chemical composition and elements in the synthesized CuS NRs. In Figure 2d, the two peaks at 932.8 and 952.9 eV matched the Cu 2p_1/2_ and Cu 2p_3/2_ valence states in the CuS NRs, respectively. In Figure 2e, the two divided peaks at 162.2 and 163.3 eV were linked with the S 2p_1/2_ and S 2p_3/2_ valence states, respectively. As shown in Figure 2f, C 1s revealed three peaks in CuS NRs at 285.2, 286.9, and 289.2 eV, matching C-C, C-C-OH, and O-C=O, respectively. The C 1s peaks were attributed to atmospheric oxidation and the absorption of atmospheric moisture to produce reactive oxygen species [55,56]. The XPS spectrum verified the production of CuS NRs. Furthermore, the FTIR spectra of CuS NRs, UC, 0.1CuS/UC, 0.3CuS/UC, 0.5CuS/UC, and 0.8CuS/UC were obtained, as shown in Figure 2g. The faint peaks at 626 cm^−1^ can be attributed to CuS. Peaks were detected at 798 cm^−1^ (–CH_3_ rocking and Si-C stretching in Si-CH_3_), 1025 cm^−1^ (Si-O-Si stretching), and 1259 cm^−1^ (CH_3_ deformation in Si-CH_3_). The presence of a peak at 3427 cm^−1^, due to hydroxyl (O–H) stretching, indicated that atmospheric moisture influenced the morphological variations in the CuS NR and CuS/UC preparations [57,58]. Overall, the structural and optical characterizations validated and confirmed the formation of CuS NRs and CuS/UC.

### 2.2. Cytotoxicity Assay of CuS NRs

**Figure 3:**
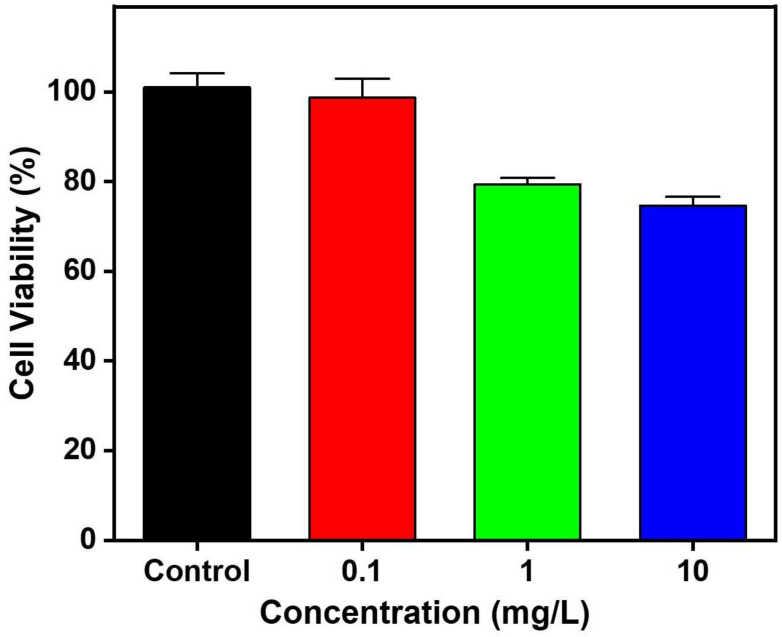
Cell viability of CuS NRs at various concentrations, including 0.1 (red), 1 (green), and 10 (blue) mg/L. Sterilized water was added as the control experiment (black).

The biological incompatibility and inherent toxicity of nanomaterials limit their biomedical applications [59]. Therefore, cytotoxicity assays are essential for determining the effects of these materials on mammalian cells. In this paper, the toxicity test of CuS NRs on Vero cells was assessed using the MTT assay, which is a widely accepted method for evaluating cell viability [16]. As depicted in Figure 3, the cell viability of CuS NRs at the concentrations of 0.1, 1, and 10 mg/L were measured to be 98.69%, 79.38%, and 74.65%, respectively. There is a noticeable decreasing trend in cell viability as Vero cells are exposed to CuS NRs. However, even at the highest concentration tested (10 mg/L), the recorded cell viability was 74.65%. Based on the guidelines outlined in the UNI EN ISO 10993/2009 standard, which offers criteria for evaluating the biocompatibility of medical devices, a sample is considered non-cytotoxic if the cell viability exceeds 70% [60]. Since the cell viability of CuS NRs remained above this threshold in this study, it can be concluded that these materials demonstrate low cytotoxicity levels and show promise for potential clinical applications.

### 2.3. Surface Studies of CuS/UC

**Figure 4:**
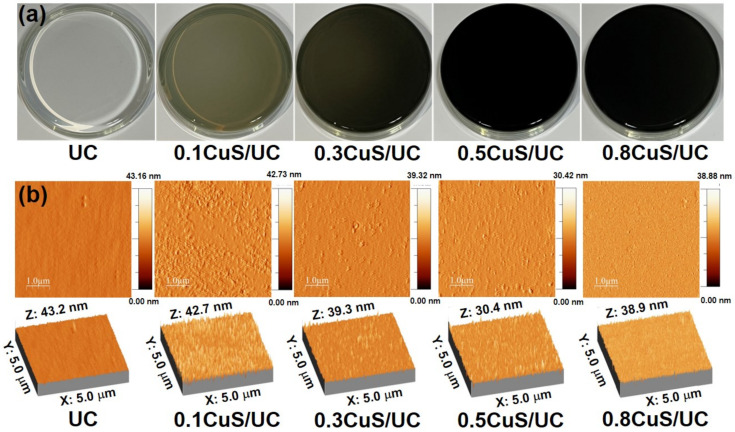
(**a**) Photos of UC, 0.1CuS/UC, 0.3CuS/UC, 0.5CuS/UC, and 0.8CuS/UC. (**b**) Two-dimensional and three-dimensional AFM images of UC, 0.1CuS/UC, 0.3CuS/UC, 0.5CuS/UC, and 0.8CuS/UC.

The UC was fully transparent, as shown in the photo in Figure 4a. For CuS NRs embedded in the UC, the color of the CuS/UC became darker with the increase in the concentration of CuS NRs. The increase in dark color was attributed to the dark color of the CuS NRs. Moreover, AFM was applied to examine the surface homogeneity of the UC and CuS/UC. In Figure 4b, the surface of the UC revealed a wrinkled pattern according to its 2D AFM image. For the CuS/UC, the grafted brushes created island structures on the surface during the drying process. As shown in the 3D AFM images in Figure 4b, the average heights of UC, 0.1CuS/UC, 0.3CuS/UC, 0.5CuS/UC, and 0.8CuS/UC were measured to be 43.2, 42.7, 39.3, 30.4, and 38.9 nm, respectively. The surface roughness showed no significant difference between UC and CuS/UC.

**Figure 5:**
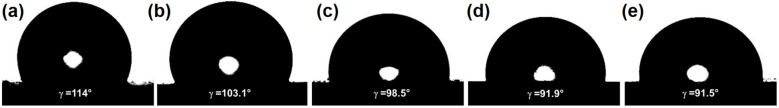
Contact angles of (**a**) UC, (**b**) 0.1CuS/UC, (**c**) 0.3CuS/UC, (**d**) 0.5CuS/UC, and (**e**) 0.8CuS/UC.

To further examine the hydrophobicity, the contact angles of the UC and CuS/UC were measured. As shown in Figure 5, the contact angles of C, 0.1CuS/UC, 0.3CuS/UC, 0.5CuS/UC, and 0.8CuS/UC were, respectively, detected to be 114°, 103.1°, 98.5°, 91.9°, and 91.5°. The contact angles of CuS/UC (at various concentrations) were smaller than that of UC due to the embedding of CuS NRs. However, the contact angles of the CuS/UC remained above 90°. These results indicate that the CuS/UC exhibited a low wettability, hydrophobicity, and low adhesiveness, making it most suitable for biomedical applications where minimal interaction with bacteria and healthy cells is required [61].

### 2.4. Photothermal Antibacterial Activity of CuS/UC

**Figure 6:**
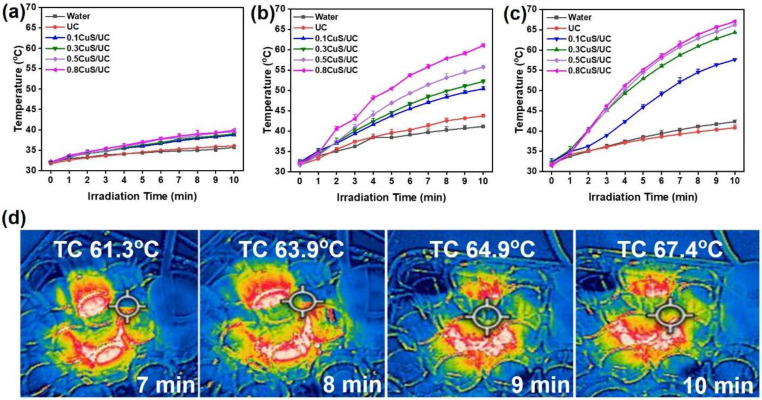
Photothermal characterizations of water, UC, 0.1CuS/UC, 0.3CuS/UC, 0.5CuS/UC, and 0.8CuS/UC with different NIR laser power densities at (**a**) 0.5 W/cm^2^, (**b**) 1.0 W/cm^2^, and (**c**) 1.5 W/cm^2^. (**d**) Real-time thermal images of 0.8CuS/UC were captured at 7 min (61.3 °C), at 8 min (63.9 °C), at 9 min (64.9 °C), and at 10 min (67.4 °C) with an exposure of NIR laser at power density of 1.5 W/cm^2^. The circles indicated the measurement points.

CuS nanomaterials have a photothermal effect due to their localized surface plasmon resonance generated by NIR irradiation [62,63]. In this work, UC, 0.1CuS/UC, 0.3CuS/UC, 0.5CuS/UC, and 0.8CuS/UC with a diameter of 15 mm were immersed in 1.5 mL of sterile water. An 808 nm NIR laser was utilized to assess the effectiveness of photothermal conversion at the power densities of 0.5, 1.0, and 1.5 W/cm^2^. In Figure 6a, with NIR laser irradiation at a power density of 0.5 W/cm^2^ for 10 min, the 0.8CuS/UC was heated to a maximal temperature of 39.9 °C. The power density of 0.5 W/cm^2^ did not adequately reach the photothermal condition for killing bacteria, as the 33~41 °C range is where the majority of pathogenic bacteria can flourish [64]. Since bacteria and biofilms have double cell walls and more energy is required to break them down, the temperature needs to be 50 to 80 °C to destroy them. Temperature elevation was aided by an increasing power density [65]. Moreover, after 10 min of NIR irradiation at a power density of 1 W/cm^2^, all samples with CuS NRs exhibited a mild temperature rise. The highest content of CuS NRs (0.8CuS/UC) reached 61.05 °C as shown in Figure 6b. For NIR laser irradiation for 10 min at a power density of 1.5 W/cm^2^, all samples with CuS NRs reached temperatures of >60 °C except the 0.1CuS/UC, as shown in Figure 6c. Moreover, the photothermal conversion efficiencies of 0.1CuS/UC, 0.3CuS/UC, 0.5CuS/UC, and 0.8CuS/UC were, respectively, calculated to be 30.67, 34.27, 34.51, and 45.18% (see Figure S2 in Supporting Information) [66,67,68]. At an irradiation intensity of 1.5 W/cm^2^, real-time 0.8CuS/UC thermal pictures were recorded at 61.3 °C at 7 min, 63.9 °C at 8 min, 64.9 °C at 9 min, and 67.4 °C at 10 min, as shown in Figure 6d. According to the photothermal characterization, the power density of 1.5 W/cm^2^ is suitable for photothermal catheter sterilization.

**Figure 7:**
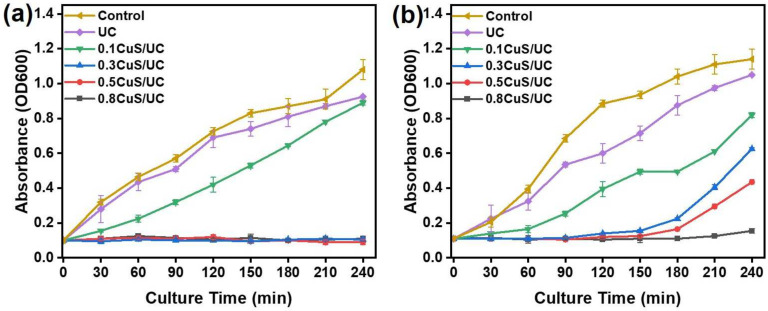
Growth curves of (**a**) *E. coli* and (**b**) *S. aureus* after irradiation with NIR laser at a power density of 1.5 W/cm^2^ for 10 min. The control samples were an *E. coli* solution without incubation with CuS/UC for (**a**) and *S. aureus* solution without incubation with CuS/UC for (**b**).

To further confirm the antibacterial activity, round-shaped CuS/UC cut samples with a 15 mm diameter were placed in 1.5 mL of a bacterial suspension and then irradiated with a 808 nm NIR laser at a power density of 1.5 W/cm^2^ for 10 min. After irradiation, the growth curves of the bacterial solutions were recorded. As shown in Figure 7a, the control sample was an *E. coli* solution with NIR irradiation and the value of OD600 was measured to be 1.08 after culture for 240 min. Compared to the control sample, the values of OD600 for UC, 0.1CuS/UC, 0.3CuS/UC, 0.5CuS/UC, and 0.8CuS/UC were, respectively, measured to be 0.925, 0.89, 0.11, 0.10, and 0.09 after culture for 240 min. The UC sample revealed a slight photothermal antibacterial activity. However, for the samples of the CuS/UC, the photothermal antibacterial activity increased with the concentration of CuS NRs embedded in the UC. Moreover, with no significant increase in the OD 600 value of *E. coli*, the 0.3CuS/UC, 0.5CuS/UC, and 0.8CuS/UC exhibited a superior photothermal antibacterial activity for *E. coli*. Furthermore, with NIR irradiation, the OD600 value of *S. aureus* was measured to be 1.14 after culture for 240 min, as shown in Figure 7b. The values of OD600 for the UC, 0.1CuS/UC, 0.3CuS/UC, 0.5CuS/UC, and 0.8CuS/UC were, respectively, obtained as 1.05, 0.82, 0.63, 0.44, and 0.16 after culture for 240 min. For *S. aureus*, the sample of the CuS/UC also demonstrated the increase in the photothermal antibacterial activity with the concentration of CuS NRs embedded in the UC. For the 0.8CuS/UC, the OD600 value of *S. aureus* only increased from 0.1 to 0.16 after culture for 240 min. The 0.8CuS/UC also provided potential for photothermal killing of *S. aureus* under NIR irradiation. Overall, the samples of the CuS/UC were demonstrated to have photothermal antibacterial activity under NIR laser irradiation. To have a better photothermal antibacterial activity, the 0.8CuS/UC was recommended due to the better photothermal conversion efficiency.

### 2.5. Evaluation of Photothermal Antibacterial Activity of CuS/UC by Agar Plate Test

**Figure 8:**
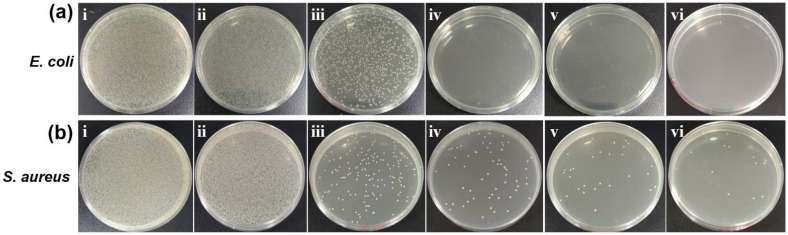
Photos of agar plates cultured with (**a**) *E. coli* and (**b**) *S. aureus*. The bacteria were treated with (**i**) control, (**ii**) UC, (**iii**) 0.1CuS/UC, (**iv**) 0.3CuS/UC, (**v**) 0.5CuS/UC, and (**vi**) 0.8CuS/UC by NIR laser irradiation for 10 min.

To evaluate the photothermal antibacterial activity, the NIR laser-induced bactericidal activities of the UC, 0.1CuS/UC, 0.3CuS/UC, 0.5CuS/UC, and 0.8CuS/UC were explored by agar plate counts. The round-shaped CuS/UC cut samples with a 15 mm diameter were placed in 1.5 mL of *E. coli* and *S. aureus* solutions. After irradiation with the NIR laser at a power density of 1.5 W/cm^2^ for 10 min, the bacterial solutions were used for the agar plate test. As shown in Figure 8a, with NIR laser irradiation, the UC exhibited a slight growth in *E. coli* according to the decrease in CFUs. For 0.1CuS/UC, 0.3CuS/UC, 0.5CuS/UC, and 0.8CuS/UC, the CFUs were significant decrease. Most importantly, for 0.3CuS/UC, 0.5CuS/UC, and 0.8CuS/UC, there were no CFUs on the agar plates. Furthermore, the bacterial survival rates of *E. coli* were calculated as shown in Table 1. The bacterial survival rates of the UC, 0.1CuS/UC, 0.3CuS/UC, 0.5CuS/UC, and 0.8CuS/UC were calculated to be 80.40, 5.55, 0.00, 0.00, and 0.00%, respectively. The 0.3CuS/UC, 0.5CuS/UC, and 0.8CuS/UC showed an outstanding photothermal killing efficiency of 100% for *E. coli*. For *S. aureus*, as shown in Figure 8b, with NIR laser irradiation, the UC treatment resulted in a slight reduction in the growth of *S. aureus*, as indicated by the decrease in CFUs. For the treatments with 0.1CuS/UC, 0.3CuS/UC, 0.5CuS/UC, and 0.8CuS/UC, the CFUs significantly decreased. However, even for the 0.8CuS/UC treatment, CFUs were still observed on the agar plates. The bacterial survival rates of *S. aureus* for the UC, 0.1CuS/UC, 0.3CuS/UC, 0.5CuS/UC, and 0.8CuS/UC were, respectively, 87.92%, 0.35%, 0.14%, 0.06%, and 0.02%, as shown in Table 1. In comparison to *E. coli*, the CuS/UC exhibited a lower photothermal antibacterial activity against *S. aureus*. The results of the agar plate test confirm the results of the bacterial growth curves. Overall, the CuS/UC demonstrated a superior photothermal antibacterial activity against both the Gram-negative *E. coli* and Gram-positive *S. aureus*.

### 2.6. Biocompatibility and In Vitro Anti-Cell Adhesion of CuS/UC

**Figure 9:**
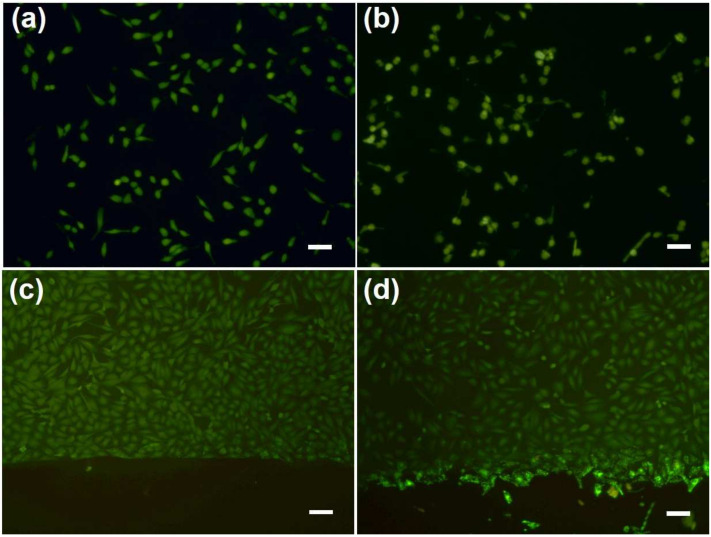
Fluorescence images of Vero Cells incubated with (**a**) UC and (**b**) 0.8CuS/UC after culture for 24 h. Fluorescence images of the boundary of (**c**) UC and (**d**) 0.8CuS/UC. All the scale bars are 100 μm.

To demonstrate the possibility for further clinical application, the biocompatibility of the CuS/UC was examined by Vero cells. As shown in the fluorescence image of Figure 9a, after culture with the UC for 24 h, the Vero cells (green) revealed normal cell proliferation. For the culture with 0.8CuS/UC for 24 h, the Vero cells also had a similar cell proliferation, as shown in Figure 9b. With normal cell proliferation, both of the UC and 0.8CuS/UC demonstrated excellent biocompatibility. The interaction between UC and Vero cells was also examined using fluorescence microscopy. After 24 h of cell culture, as shown in Figure 9c, living cells were seen gathering near the boundary of the UC, with no Vero cells appearing on the surface of the UC. Similarly, as shown in Figure 9d, living cells also gathered near the boundary of 0.8CuS/UC, with no significant adhesion of Vero cells on the surface of the 0.8CuS/UC. The anti-cell adhesion of the UC and 0.8CuS/UC was attributed to their surface hydrophobicity. These results suggest a preliminary conclusion that the 0.8CuS/UC exhibits excellent biocompatibility and anti-cell adhesion properties.

## 3. Materials and Methods

### 3.1. Chemicals

Copper (II) nitrate (Cu(NO_3_)_2_·3H_2_O, 99%) and thiourea (SC(NH_2_)_2_, 99%) were purchased from Across Organic (Morris, NJ, USA). Polydimethylsiloxane (PDMS) Sylgard^TM^ 184 silicon elastomer was purchased from Dow Silicones Corporation (Midland, MI, USA). LB broth Miller was purchased from BioShop (Burlington, ON, Canada). Bacteriological-grade agar and phosphate-buffered saline (PBS) were purchased from Bioman Scientific (Taipei, Taiwan). Trypticase soy broth (TSB) was purchased from Condalab (Madrid, Spain). Ethanol, acetone, and n-hexane were purchased from Merck (Darmstadt, Germany). Dulbecco’s Modified Eagle Medium (DMEM), fetal bovine serum (FBS), and Invitrogen Calcein AM solution were purchased from ThermoFisher Scientific (Carlsbad, CA, USA).

### 3.2. Synthesis of CuS NRs

The CuS NRs were derived from CuS nanoparticles via the hydrothermal method. CuS nanoparticles were synthesized according to our previous work with modifications [42]. Briefly, copper nitrite (0.96 g) and thiourea (0.91 g) were dissolved in 40 mL of ethanol under stirring at 5000 rpm for 30 min. The solution was then placed in an autoclave reactor and heated to 150 °C for 24 h. After 24 h, the solution was centrifuged at 7500 rpm for 10 min to separate the supernatant and precipitate. The precipitate was washed twice with deionized (DI) water and once with ethanol, and then dried at 60 °C in an oven to obtain CuS nanoparticles. The CuS nanoparticles were then sonicated in a hexane solution for 30 min to obtain CuS NRs.

### 3.3. Preparation of CuS NR-Embedded Urinary Catheter

The CuS NRs were sonicated in a hexane solution for 30 min to avoid agglomeration. The CuS NR-embedded urinary catheters were prepared by adding 7 g of Sylgard^TM^ 184 silicone elastomers with various weight percentages of CuS NRs, including 0.0% (UC), 0.1% (0.1CuS/UC), 0.3% (0.3CuS/UC), 0.5% (0.5CuS/UC), and 0.8% (0.8CuS/UC). The curing agent was slowly added and then stirred for 30 min. The homogenous solution was poured into a 90 mm diameter glass Petri dish to obtain CuS NR-embedded urinary catheters. A vacuum oven was used to remove bubbles in the CuS NR-embedded urinary catheters at 40 °C for 30 min. Afterward, the temperature of the vacuum oven was increased to 70 °C for the curing process for 12 h.

### 3.4. Material Characterization

The morphology and EDX analysis of CuS NRs were conducted by SEM (SU-3500, Hitachi, Tokyo, Japan), TEM (HT-7700, Hitachi, Tokyo, Japan), and HR-TEM (JEM-2100F, JEOL, Tokyo, Japan). The Raman spectra of CuS NRs were obtained by laser spectroscopy confocal micro-Raman spectroscopy (UniDRON, Taoyuan, Taiwan). The elemental composition of the CuS NRs was studied by XPS (ESCALAB 250, VG Scientific, Waltham, MA, USA). The crystalline structures of the CuS NRs and CuS/UC were characterized by XRD with CuKα radiation (D2 PHASER, Bruker, Billerica, MA, USA), and phase analysis was conducted using X’Pert HighScore Plus 2.0a version equipped with data from the PDF2 database. The absorption spectra of the CuS NRs and CuS/UC were recorded by UV-Vis-NIR spectrophotometry (JASCO-V770, JACSO, Tokyo, Japan). The functional groups of the CuS NRs, UC, and CuS/UC were validated by Fourier-transform infrared spectroscopy (FTIR, Nicolet iS50, Thermo Fisher Scientific, MA, USA). The surface morphology was investigated by AFM (FSM-Nanoview1000, UTEK MATERIAL, Taipei, Taiwan). The contact angle was measured with a DIGIDROP instrument (Dublin, Ireland).

### 3.5. Photothermal Properties of CuS/UC

A NIR laser at 808 nm was used as the light source to trigger the photothermal properties of CuS/UC. Each sample was cut into a round shape with a diameter of 15 mm and placed in a culture tube with 1.5 mL of sterile water. Afterward, the samples were irradiated using different NIR laser energy densities of 0.5, 1.0, and 1.5 W/cm^2^ for 10 min and recorded every 60 s with a thermal imaging camera (FLIR TG267, Wilsonville, OR, USA) equipped with a thermocouple cable. Photothermal data were used as references for deciding the power density and time irradiation for bacterial sterilization.

### 3.6. Photothermal Antibacterial Assay

*S. aureus* (ATCC 25923) and *E. coli* (BL21(DE3)) were cultured in TSB medium, with rotation at 170 rpm in an incubator at 37 °C overnight to obtain an OD600 (optical density at 600 nm) value of 1.25. Then, the bacterial suspension was diluted with TSB solution until an OD600 value of 0.1 was recorded. The round-shaped samples with a 15 mm diameter of UC, 0.1CuS/UC, 0.3CuS/UC, 0.5CuS/UC, and 0.8CuS/UC were placed in a culture tube with 1.5 mL of the bacterial suspension. The samples in the bacterial solutions were then irradiated with NIR laser at energy density of 1.5 W/cm^2^ for 10 min. Afterward, the bacterial solutions were placed in an incubator shaker at 170 rpm and 37 °C, and the growths of the bacteria were recorded every 30 min for 3 h. Furthermore, 20 µL of the irradiated bacterial solution was taken out, seeded onto agar plates, and placed in an incubator at 37 °C for 24 h. The bactericidal performance was calculated based on Equation (1):

(1) $$Bacteria\;Killing\;Ratio=\frac{N_{control}-N_{sample}}{N_{control}}\;\times \;100\%$$

where N_control_ and N_sample_ are the colony-forming units (CFUs) of the UC and CuS/UC with NIR laser irradiation, respectively. All the experiments were repeated three times.

### 3.7. Cytotoxicity of CuS NRs

The MTT assay was used to evaluate the cytotoxicity of the CuS NRs. In particular, Vero cells were seeded on a 98-well plate at a density of 7.5 × 10^3^/well cultured in DMEM, supplemented with 10% FBS and 1% antibiotic–antimycotic solution, and incubated in 5% CO_2_ at 37 °C. Before the Vero cells were treated with the CuS NRs, the culture medium was changed to a starvation medium (0.1% FBS) for 16 h and then cultivated with various concentrations of CuS NRs (0.1, 1, and 10 mg/L) for 48 h. After culture, all medium was removed, and 100 μL of MTT solution (2 mg/mL) was added to each well and incubated for 4 h. Then, 100 μL of DMSO was added to each well, shaking for 1 h to ensure complete solubilization. The absorbance of OD570 was used for determining the cell viability by following Equation (2).

(2) $$Cell\;viability\left(\%\right)=\frac{{OD}_{sample}-{OD}_{blank}}{{OD}_{control}}\;\times \;100\%$$

where OD_sample_, OD_blank,_ and OD_control_ are the absorbances of the cells with CuS NRs, culture media, and the cells without CuS NRs, respectively [69]. All the experiments were repeated three times.

### 3.8. Biocompatibility Test of CuS/UC

An in vitro assay was used to examine the biocompatibility of the UC and CuS/UC samples. The UC and CuS/UC with 15 mm diameter were placed on a 6-well culture plate and then incubated with the Vero cells at a density of 6 × 10^5^ cells/well for 24 h. After incubation, the cells were stained with 1.5 µL of 1 mM Calcein AM in a culture medium and further incubated for 1 h at 37 °C to identify the live cells. After that, the culture medium was removed, the solution (1× PBS) was used to wash the cells twice, and then, 1 mL of 1× PBS solution was added to the cells. Finally, the proliferation of the Vero cells was observed by fluorescence microscopy.

## 4. Conclusions

In summary, CuS NRs were successfully synthesized via the hydrothermal method. The CuS NRs demonstrated low cytotoxicity, suggesting potential for clinical applications. These CuS NRs were embedded into urinary catheters to form CuS/UC with various weight percentages, including 0.1CuS/UC, 0.3CuS/UC, 0.5CuS/UC, and 0.8CuS/UC. The surface roughness showed no significant difference between the UC and CuS/UC. With a contact angle above 90°, the CuS/UC exhibited a low wettability, hydrophobicity, and low adhesiveness, making them highly suitable for biomedical applications requiring minimal interaction with bacteria and healthy cells. Under NIR laser irradiation at 1.5 W/cm² for 10 min, the 0.8CuS/UC reached a temperature of 67.4 °C, indicating its suitability for photothermal catheter sterilization. The samples of the CuS/UC demonstrated photothermal antibacterial activity under NIR laser irradiation, with 0.8CuS/UC being recommended for its superior photothermal conversion efficiency. The agar plate test confirmed that the CuS/UC exhibited a superior photothermal antibacterial activity against both Gram-negative *E. coli* and Gram-positive *S. aureus*. The properties of photothermal performance, excellent biocompatibility, and anti-cell adhesion of the 0.8CuS/UC demonstrated its potential as a photothermal antibacterial catheter for clinical applications.

## Figures and Tables

**Table 1:** Bacterial survival rates.

Bacteria	Control	UC	0.1CuS/UC	0.3CuS/UC	0.5CuS/UC	0.8CuS/UC
*E. coli*	100	80.40	5.55	0.00	0.00	0.00
*S. aureus*	100	87.92	0.35	0.14	0.06	0.02

## Data Availability

All data are contained within the article.

## Supplementary Materials

The supporting information can be downloaded at: https://www.mdpi.com/article/10.3390/ijms252111440/s1.
