# Supplementary material for: Copper Sulfide Nanorod-Embedded Urinary Catheter with Hydrophobicity and Photothermal Sterilization
Source: Int J Mol Sci. 2024 Oct 24;25(21):11440. doi: 10.3390/ijms252111440 (PMC11546957; doi:10.3390/ijms252111440)
Supplement: Supplementary file 1 [file ijms-25-11440-s001.zip › ijms-3268064-supplementary.pdf]

## Supporting Information

# Copper Sulfide Nanorod-Embedded Urinary Catheter with Hydrophobicity and Photothermal Sterilization

**Muhammad Saukani [1,2,†], Chien-Hung Lai [3,4,5,†], Chinmaya Mutalik [6], Dyah Ika Krisnawati [7], Hsiu-Yi Chu [8] and Tsung-Rong Kuo[1,6,*]**

[1] International Ph.D. Program in Biomedical Engineering, College of Biomedical Engineering, Taipei Medical University, Taipei 11031, Taiwan; d845110002@tmu.edu.tw
[2] Department of Mechanical Engineering, Faculty of Engineering, Universitas Islam Kalimantan MAB, Banjarmasin 70124, Kalimantan Selatan, Indonesia
[3] Department of Physical Medicine and Rehabilitation, School of Medicine, College of Medicine, Taipei Medical University, Taipei 11031, Taiwan; chlai@tmu.edu.tw
[4] Department of Physical Medicine and Rehabilitation, Taipei Medical University Hospital, Taipei 11031, Taiwan
[5] Taipei Neuroscience Institute, Taipei Medical University, Taipei 11031, Taiwan
[6] Graduate Institute of Nanomedicine and Medical Engineering, College of Biomedical Engineering, Taipei Medical University, Taipei 11031, Taiwan; cm121193@tmu.edu.tw
[7] Department of Nursing, Faculty of Nursing and Midwifery, Universitas Nahdlatul Ulama Surabaya, Surabaya 60237, East Java, Indonesia; dyahkrisna77@gmail.com
[8] Graduate Institute of Biomedical Materials and Tissue Engineering, College of Biomedical Engineering, Taipei Medical University, Taipei 11031, Taiwan; d825111001@tmu.edu.tw

* Correspondence: trkuo@tmu.edu.tw
† These authors contributed equally to this work.

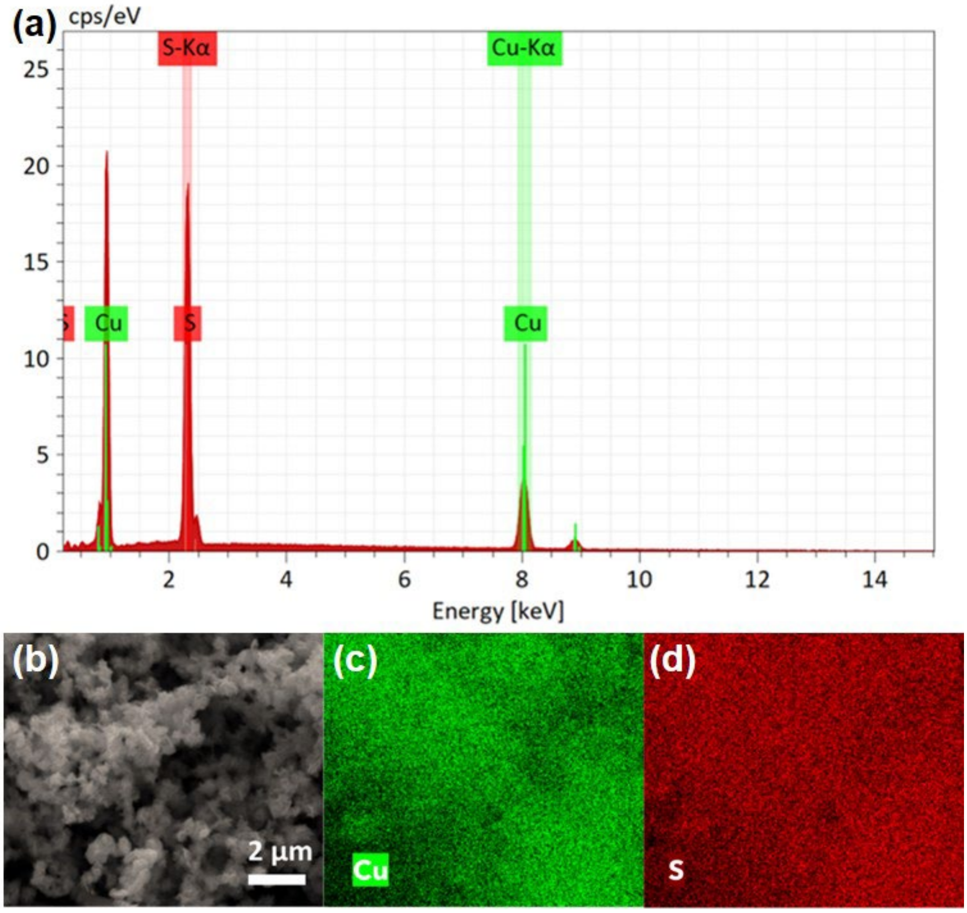


**Figure S1.** (a) EDX analysis of CuS NRs. (b) SEM image of CuS NRs. The corresponding elemental mapping of (c) Cu and (d) S.

## Calculation of Photothermal Conversion Efficiency of the CuS/UC

The photothermal conversion efficiency of the CuS/UC was determined using a previously established method.[1-3] The detailed calculation was as follows:

$$\eta = \frac{hS\,(T_{max} - T_{surr}) - Q_{dis}}{I(1 - 10^{-A_{808}})} \qquad (1)$$

In the equation (1), $h$ is the heat transfer coefficient, $S$ represents the surface area for heat transfer, $T_{max}$ is the equilibrium temperature, and $T_{surr}$ indicates the

temperature of the surrounding environment. $Q_{dis}$ is the heat dissipated to the surrounding environment, $I$ is the intensity of the laser irradiation, and $A_{808}$ is the absorbance of the photothermal material at wavelength 808 nm. In order to obtain *hS*, a parameter $\theta$ is conducted as followed:

(2)

The time constant of a sample system can be obtained by equation (3).

$$\tau_s = \frac{t}{-\ln(\theta)} \qquad (3)$$

The values of *hS* can be calculated by the equation (4).

$$hS = \frac{m_D\, C_D}{\tau_s} \qquad (4)$$

For example, based on above approach, the photothermal conversion efficiency of the 0.8CuS/UC was calculated. From the experimental design, the $T_{surr}$ was 24.5 °C, the laser intensity is 1.5 W, and the $Q_{dis}$ was 11.28 J. The absorbance of 0.8CuS/UC was 1.56 at wavelength of 808 nm. From Figure S1a, the $T_{max}$ was 70.1 °C. From Figure S2b, $\tau_s$ was calculated to be 284.34 s and then *hS* was obtained as 13.30 mW/°C. Therefore, the photothermal conversion efficiency of 0.8CuS/UC was calculated to be 45.18%. For 0.1CuS/UC, 0.3CuS/UC, and 0.5CuS/UC, photothermal conversion efficiencies were calculated to be 30.67, 34.27, and 34.51, respectively.

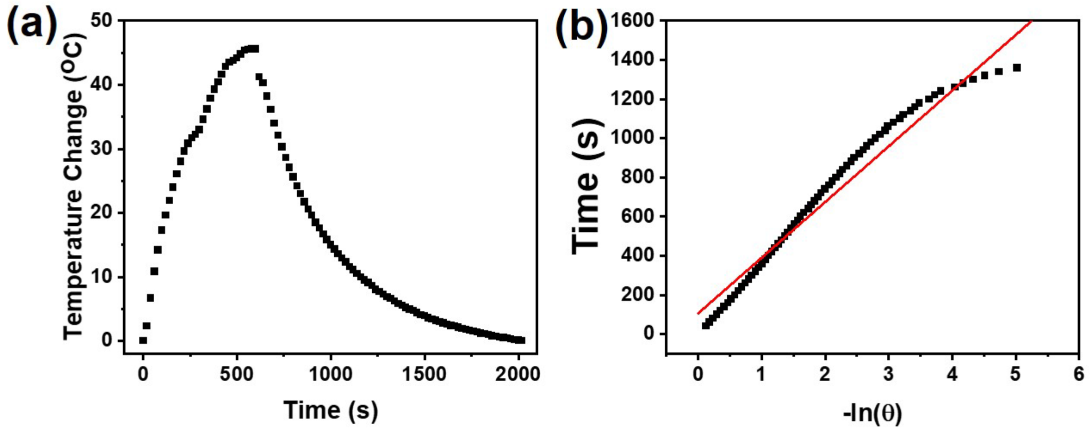


**Figure S2.** (a) Photothermal curve of 0.8CuS/UC with 808 nm laser irradiation. (b) Linear time data versus -ln(θ) obtained from the cooling period of Figure S2a.

## References


1. Cui X., Ruan, Q., Zhuo, X., Xia, X., Hu, J., Fu, R., Li, Y., Wang, J., Xu, H. Photothermal nanomaterials: A powerful light-to-heat converter. *Chem. Rev.* **2023,** *123*, 6891-6952.
2. Chen H., Shao, L., Ming, T., Sun, Z., Zhao, C., Yang, B., Wang, J. Understanding the photothermal conversion efficiency of gold nanocrystals. *Small* **2010,** *6*, 2272-2280.
3. Marin R., Skripka, A., Besteiro, L. V., Benayas, A., Wang, Z., Govorov, A. O., Canton, P., Vetrone, F. Highly efficient copper sulfide-based near-infrared photothermal agents: Exploring the limits of macroscopic heat conversion. *Small* **2018,** *14*, 1803282.
